# TLR4 promoted endoplasmic reticulum stress induced inflammatory bowel disease via the activation of p38 MAPK pathway

**DOI:** 10.1042/BSR20220307

**Published:** 2022-04-21

**Authors:** Tian Hu, Yan Zhao, Yan Long, Xiaoqing Ma, Ya Zeng, Weijie Wu, Chongtian Deng, Mengling Li, Siyuan Peng, Hanzhi Yang, Mi Zhou, Jinyue Hu, Yueming Shen

**Affiliations:** 1 Department of Digestive Diseases, Changsha Central Hospital, No. 161 Shaoshan Nanlu, Changsha, Hunan; 2 Department of Pathology, Changsha Central Hospital, No. 161 Shaoshan Nanlu, Changsha, Hunan; 3 Zhongshan City People Hospital, No. 2, Sunwen East Road, Zhongshan, Guangdong; 4 Central Laboratory, Changsha Central Hospital, No. 161 Shaoshan Nanlu, Changsha, Hunan

**Keywords:** inflammatory bowel disease, mitogen-activated protein kinases, tlr4

## Abstract

Endoplasmic reticulum (ER) stress contribute to inflammatory bowel disease (IBD). However, the mechanistic link between toll-like receptor 4 (TLR4) and ER stress in IBD remains elusive. This study aimed to investigate the mechanism by which ER stress and TLR4 promote inflammation in IBD.

IBD mouse model was established by the induction of TNBS, and Grp78 and TLR4 in intestine tissues were detected by immunohistochemistry. THP-1 cells were treated with lipopolysaccharides (LPS), ER stress inducer or inhibitor tauroursodeoxycholic acid (TUDCA), or p38 MAPK inhibitor. The activation of MAPK signaling was detected by Western blot, and the production and secretion of inflammatory factors were detected by PCR and ELISA. We found that the expression levels of TLR4 and GRP78 were significantly higher in the intestine of IBD model mice compared with control mice but were significantly lower in the intestine of IBD model mice treated with ER stress inhibitor TUDCA. ER stress inducer significantly increased while ER stress inhibitor TUDCA significantly decreased the expression and secretion of TNF-α, IL-1β and IL-8 in THP-1 cells treated by LPS. Only p38 MAPK signaling was activated in THP-1 cells treated by ER stress inducer. Furthermore, p38 inhibitor SB203580 inhibited the production and secretion of TNF-α, IL-1β and IL-8 in THP-1 cells treated with LPS. In conclusion, TLR4 promotes ER stress induced inflammation in IBD, and the effects may be mediated by p38 MAPK signaling. TLR4 and p38 MAPK signaling are novel therapeutic targets for IBD.

## Introduction

Inflammatory bowel disease (IBD) is a chronic gastrointestinal inflammatory disorder [1]. Although gut microbiome dysbiosis, host immune response, environmental and genetic factors have been implicated in the development of IBD, the pathogenesis of IBD remains largely unclear [2–4]. Notably, recent evidence suggests that endoplasmic reticulum (ER) stress and toll-like receptor 4 (TLR4) contribute to the development of IBD [5,6].

Various cellular stress conditions cause the accumulation of unfolded or misfolded proteins in the ER lumen, leading to ER stress [7,8]. The accumulation of unfolded and misfolded proteins in the ER lumen activates unfolded protein response (UPR) to repair protein folding defect and restore ER homeostasis. GRP78 is a key regulator of ER stress because it is a major ER chaperone with antiapoptotic activity and it controls the activation of UPR signaling [9]. When ER stress is serious and can not be alleviated by UPR, inflammation will develop [7].

Toll-like receptors (TLRs) play important role in recognizing pathogens and activating innate immune [10]. TLR4 is the first identified TLR that recognizes lipopolysaccharides (LPS) of Gram-negative bacteria and activates inflammation via MAL-myeloid differentiation factor 88 (MyD88) dependent and independent signaling pathways [11]. TLR4 signaling has been shown to contribute to intestine inflammation [12]. However, the link between TLR4 and ER stress in the development of IBD and the underlying signaling pathways are unclear. Therefore, this study aimed to investigate the mechanism by which ER stress and TLR4 promote inflammation in IBD.

## Methods

### Chemicals

Trinitrobenzene sulphonic acid (TNBS), tauroursodeoxycholic acid (TUDCA), lipopolysaccharides (LPS), tunicamycin (TM) and thapsigargin (TG) were purchased from Sigma (St louis, MO, U.S.A.). All antibodies were purchased from Abcam (Cambridge, U.K.).

### Animals

All animal experiments were performed at Changsha Central Hospital (Changsha, China), and all protocols were approved by the Institutional Animal Care Committee of Changsha Central Hospital. Specific pathogen free BALB/c mice (female 6–8 weeks old, weight 18–22 g) were purchased from Hunan Slake Jingda Laboratory Animal Ltd (Changsha, China). The mice were acclimatized for 1 week and maintained at 22°C under a 12-h day/night cycle.

### Experimental design

Mouse IBD model was established by the induction with TNBS following previous protocol [13]. Briefly, 75 μl of TNBS (5% w/v) and 75 μl of ethanol absolute were fully mixed to form 130 μl of TNBS enema. After fasting for 24 h, the mice were anesthetized by intraperitoneal injection with 10% chloral hydrate (3 ml/kg). Next, Intragastric needle was slowly inserted into the intestinal cavity through the anus and TNBS enema was injected. The mice were inverted for about 5 min to ensure that the enema fluid was fully dispersed in the intestinal cavity. The model mice then received intraperitoneal injection of TUDCA at dose of 50 mg/kg or normal saline (NS) as the control every day from the 2nd day after enema. Disease activity index (DAI) score was calculated as the sum of the weight loss score, the diarrheal score and the hematochezia score as described previously [13]. At the end, all mice were killed by cervical dislocation.

### Immunohistochemical staining

Intestine tissues were dissected from the mice, embedded in paraffin and cut into sections. Next, the sections were boiled in 10 mM sodium citrate buffer for antigen retrieval. After blocking endogenous peroxidase with 3% hydrogen peroxidase, sections were blocked for 30 min with normal serum, and then incubated with antibodies for GRP78 and TLR4 (R&D system) for 24 h at 4°C, and horseradish peroxidase labeled secondary antibodies. The sections were observed under microscope.

### Cell culture

Human monocytic leukemia cell line THP-1 was purchased from American Type Culture Collection and cultured in RPMI 1640 medium (Invitrogen) supplemented with 10% fetal bovine serum (FBS, Invitrogen), 10,000 U/ml penicillin and 10,000 µg/ml streptomycin in a humidified incubator with 5% CO_2_ at 37°C. Cells were treated with LPS to induce inflammation. In addition, cells were treated with TM or TG to induce ER stress [14]. Control cells were treated with dimethyl sulfoxide (DMSO) as the vehicle.

### Western blot analysis

THP-1 cells were lysed in RIPA lysis buffer containing 1% phenylmethane sulfonyl fluoride (PMSF) for 30 min on ice, and centrifuged at 12,000 rpm for 5 min at 4°C. Next, protein samples were separated in 8% sodium dodecylsulfate-polyacrylamide gel by electrophoresis and transferred on to polyvinylidene fluoride membrane. The membrane was blocked with 3% skim milk solution in phosphate buffered saline containing 0.1% (v/v) Tween-20 (PBST) for 1 h at room temperature, and incubated with primary antibodies for GRP78, TLR4, p-JNK, p-ERK1/2, p-P38, p-NF-κB-p65 and GAPDH overnight at 4°C. The membrane was then incubated with horseradish peroxidase-conjugated secondary antibodies at room temperature for 1 h, developed with enhanced chemiluminescence (ECL) kit and exposed to X-ray films. The protein bands were quantified by using Image Pro Plus 6.0 software.

### Enzyme-linked immunosorbent assay (ELISA)

The supernatants were collected from THP-1 cells, and TNF-α, IL-1β and IL-8 levels in the supernatants were measured using ELISA kits (R&D system) following the manufacturer’s protocols.

### Statistical analysis

Statistical analyses were performed using GraphPad Prism 8.0, and all values were presented as means ± SD. The differences between groups were compared by Student’s *t*-test. *P*<0.05 was considered significant.

## Results

### Up-regulation of ER stress marker and TLR4 in intestinal tissues of IBD model

**Figure 1:**
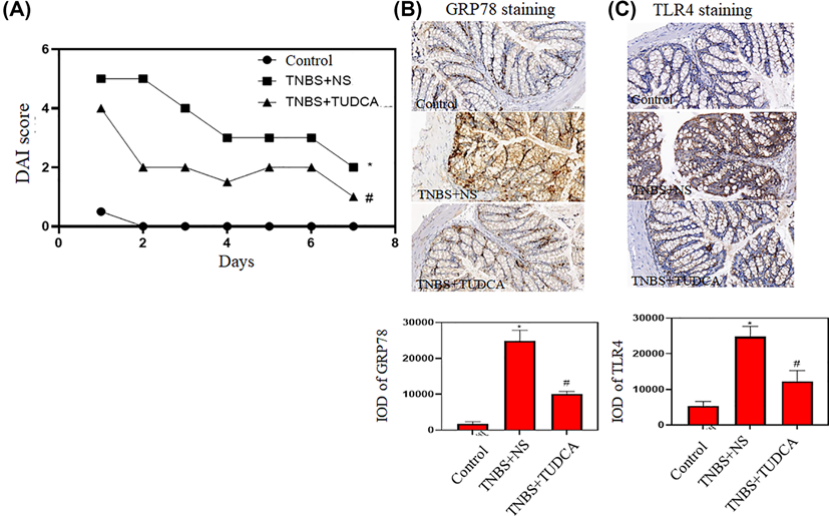
Up-regulation of ER stress marker and TLR4 in intestinal tissues of IBD model (**A**) DAI scores in three groups of mice (*n*=5). (**B**) Immunohistochemical analysis of GRP78 in the intestine of three groups of mice. (**C**) Immunohistochemical analysis of TLR4 in the intestine of three groups of mice. Staining intensity was quantified by ImageJ software and illustrated as bar charts (*n*=5). *P<0.05 compared with control. # *P*<0.05 compared with TNBS+NS.

To confirm that we successfully establish IBD mouse model, we examined DAI score in three groups of mice. Compared with control mice which had DAI score of almost 0, DAI score was significantly higher in TNBS model mice treated with NS as the control. After treatment with ER stress inhibitor TUDCA, DAI score in TNBS model mice was significantly lower (Figure 1A). These results indicated that TNBS induced intestine inflammation in the mice and intestine inflammation could be inhibited by ER stress inhibitor.

Next we examined ER stress marker GRP78 in three groups of mice. Immunohistochemical analysis showed that GRP78 staining was weak in the intestine of control mice and was strong in the intestine of TNBS model mice treated with NS as the control. After treatment with ER stress inhibitor TUDCA, GRP78 staining in TNBS model mice was significantly weaker (Figure 1B). These results indicated that TNBS induced ER stress in the intestine of the mice and ER stress was relieved by ER stress inhibitor.

Furthermore, we examined TLR4 expression in three groups of mice. Immunohistochemical analysis showed that TLR4 staining was weak in the intestine of control mice and was strong in the intestine of TNBS model mice treated with NS as the control. After treatment with ER stress inhibitor TUDCA, TLR4 staining in TNBS model mice was significantly weaker (Figure 1C). These results indicated that TNBS induced inflammation in the intestine of the mice and the inflammation was relieved by ER stress inhibitor.

### ER stress promoted LPS induced secretion of inflammatory factors in THP-1 cells

**Figure 2:**
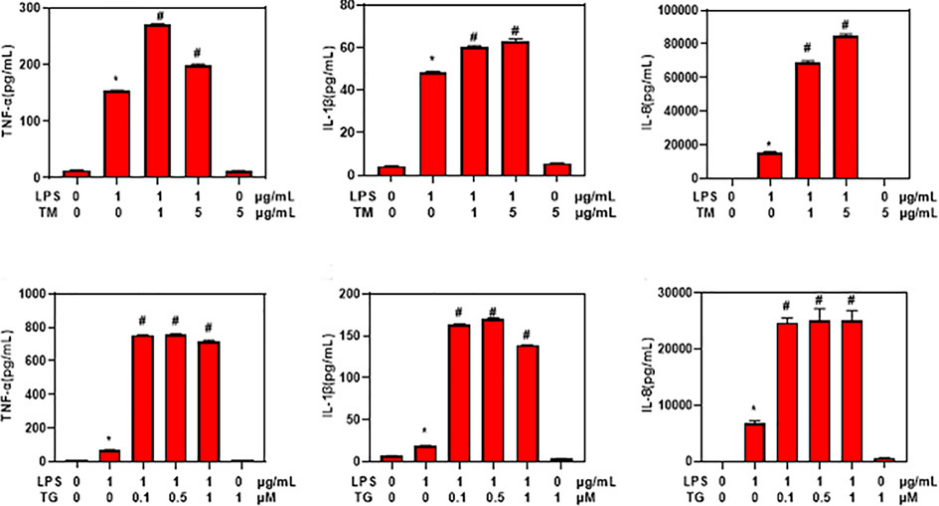
ER stress promoted LPS induced production and secretion of inflammatory factors in THP-1 cells ELISA assay of TNF-α, IL-1β and IL-8 levels in the supernatants of THP-1 cells treated with LPS, TM, TG. Values were expressed as mean ± SD (*n*=5). *P<0.05 compared with untreated control cells. #*P*<0.05 compared with cells treated with LPS alone.

To identify the potential relationship of ER stress and inflammation, THP-1 cells were treated with LPS to induce inflammation and were treated with different concentrations of TM and TG to induce ER stress. ELISA assay showed that combined treatment with LPS and TM or TG showed same trend of TNF-α, IL-1β and IL-8 (Figure 2). These results indicated that ER stress could promote LPS induced secretion of inflammatory factors *in vitro.*

### ER stress inhibitor inhibited LPS induced secretion of inflammatory factors in THP-1 cells

**Figure 3:**
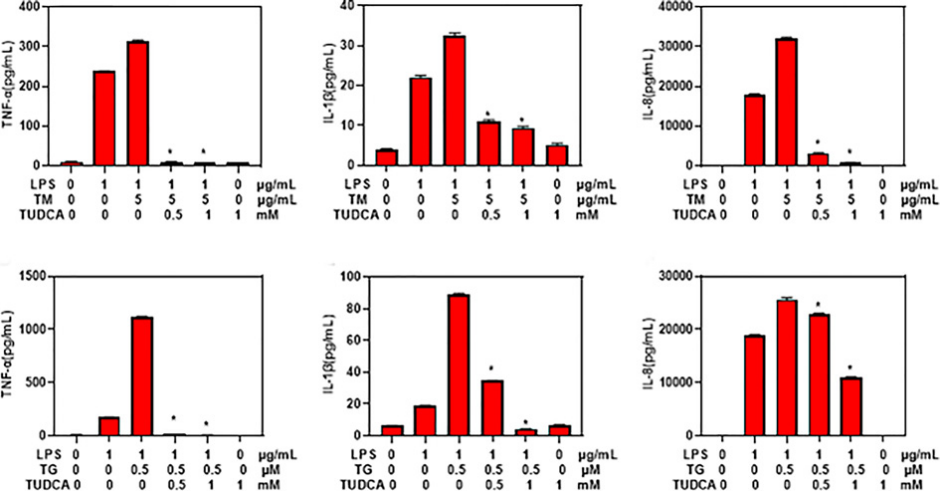
ER stress inhibitor inhibited LPS induced production and secretion of inflammatory factors in THP-1 cells ELISA assay of TNF-α, IL-1β and IL-8 levels in the supernatants of THP-1 cells treated with LPS, TM, TG, TUDCA. Values were expressed as mean ± SD (*n*=5). *P<0.05 compared with cells treated with LPS and TM or TG.

To confirm the role of ER stress in the promotion of inflammation, THP-1 cells were additionally treated with ER stress inhibitor TUDCA. ELISA assay showed that TUDCA significantly decreased the secretion of TNF-α, IL-1β and IL-8 in THP-1 cells treated with LPS, TM and TG (Figure 3). These results confirmed that ER stress could promote LPS induced secretion of inflammatory factors *in vitro*.

### p38 MAPK signaling contributed to TM or TG induced ER stress in THP-1 cells

**Figure 4:**
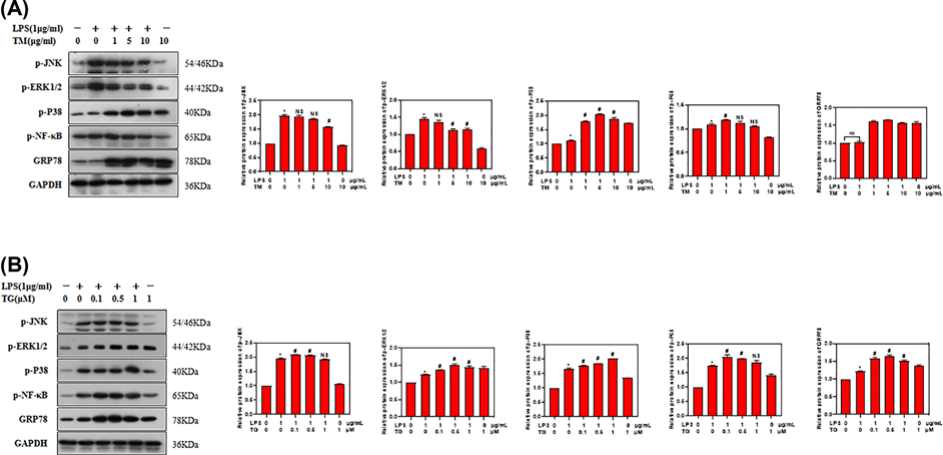
p38 MAPK signaling contributed to TM or TG induced ER stress in THP-1 cells (**A**) Western blot analysis of p-JNK, p-ERK1/2, p-p38, NF-κB-p65 and GRP78 in THP-1 cells treated with LPS and TM. (**B**) Western blot analysis of p-JNK, p-ERK1/2, p-p38, NF-κB-p65 and GRP78 in THP-1 cells treated with LPS and TG. GAPDH was loading control. Values were expressed as mean ± SD (*n*=5). *P<0.05 compared with untreated control cells. NS (not significant) compared with cells treated with LPS alone. #*P*<0.05 compared with cells treated with LPS alone.

To identify signaling pathway responsible for the promotion of inflammation by ER stress, we detected the activation/phosphorylation of JNK, ERK1/2, p38, NF-κB-p65 in THP-1 cells treated with LPS, TM and TG. Western blot analysis showed that only the levels of p-p38 increased with increasing concentrations of TM or TG, while the levels of p-JNK and p-ERK1/2 did not further increase with increasing concentrations of TM or TG. Consistently, the levels of ER stress marker GRP78 and inflammation marker p-NF-κB-p65 increased after treatment with TM or TG (Figure 4A,B, Supplementary File). Collectively, these data indicated that p38 MAPK signaling contributed to TM or TG induced ER stress and inflammation in THP-1 cells.

### p38 inhibitor inhibited LPS induced secretion of inflammatory factors in THP-1 cells

**Figure 5:**
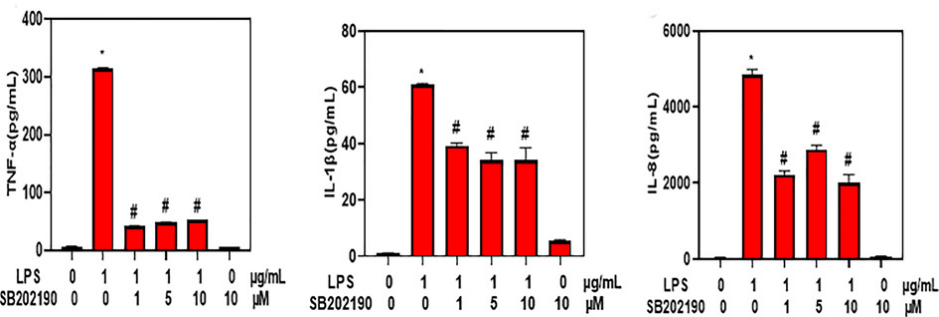
p38 inhibitor inhibited LPS induced secretion of inflammatory factors in THP-1 cells ELISA assay of TNF-α, IL-1β and IL-8 levels in the supernatants of THP-1 cells treated with LPS and SB203580. Values were expressed as mean ± SD (*n*=5). *P<0.05 compared with untreated control cells. #*P*<0.05 compared with cells treated with LPS alone.

To confirm the role of p38 MAPK signaling in the promotion of inflammation, THP-1 cells were treated with p38 MAPK kinase inhibitor SB203580. ELISA assay showed that SB203580 significantly decreased the secretion of TNF-α, IL-1β and IL-8 in THP-1 cells treated with LPS (Figure 5). These results confirmed that p38 MAPK signaling could promote LPS induced secretion of inflammatory factors *in vitro*.

## Discussion

The exposure to LPS is linked to IBD. LPS can bind to TLR4 on the surface of immune cells, and activate downstream signaling to initiate the production and secretion of inflammatory factors such as TNF-α, IL-1β and IL-8. These will lead to the development of IBD [15]. On the other hand, ER stress is involved in IBD. The inhibitors of ER stress such as TUDCA led to improved symptoms in IBD mouse model, accompanied by reduced levels of interleukin-1β (IL-1β), interferon-γ (IFN-γ) and tumour necrosis factor-α (TNF-α) in intestine tissues [16]. Therefore, it is important to understand how ER stress contributes to IBD via the activation of TLR4 signaling.

In this study, we reported that both TLR4 and ER stress played significant role in intestinal inflammation. By establishing TNBS induced IBD mouse model, we demonstrated that the expression levels of TLR4 and ER stress marker GRP78 were significantly higher in the intestine of IBD model mice compared with control mice but were significantly lower in the intestine of IBD model mice treated with ER stress inhibitor TUDCA. These results suggest that TLR4 and ER stress are closely related in IBD.

To reveal the mechanistic link between TLR4 and ER stress, we employed THP-1 cells as the experimental model. We found that ER stress inducer significantly increased the secretion of TNF-α, IL-1β and IL-8 in THP-1 cells treated by LPS, while ER stress inhibitor TUDCA significantly decreased the secretion of TNF-α, IL-1β and IL-8. These results suggest that ER stress may promote LPS induced inflammation, consistent with previous report that ER stress is involved in inflammation in IBD [17]. In addition, TUDCA was shown to inhibit ER stress and colon inflammation in mice [18].

MAPK signaling pathways play important role in the response to ER stress [19,20]. To elucidate signaling pathway that may mediate the promotion of inflammation by ER stress, we examined JNK, ERK1/2 and p38 MAPK signaling pathways and found that only p38 MAPK signaling was activated in THP-1 cells treated by ER stress inducer. The detection of increased levels of GRP78 and p-NF-κB-p65 confirmed the induction of ER stress and inflammation in these cells. Furthermore, we employed p38 MAPK kinase inhibitor SB203580 to treat THP-1 cells. ELISA assay showed that SB203580 inhibited the production and secretion of inflammatory factors such as TNF-α, IL-1β and IL-8 in THP-1 cells treated with LPS. Both TM and TG are used to induce ER stress, but they have different mechanism. TG induces ER stress by irreversibly inhibiting the sarco/endoplasmic reticulum Ca2+-ATPase (SERCA), while TM inhibits N-linked glycosylation [21]. The different mechanism may account for the difference in ER stress induced inflammation. Taken together, these results suggest that p38 MAPK signaling may mediate LPS induced inflammation.

In conclusion, our findings indicate that TLR4 promotes ER stress induced inflammation in IBD, and the effects may be mediated by p38 MAPK signaling. TLR4 and p38 MAPK signaling are novel therapeutic targets for IBD.

## Data Availability

All data and material are included in this manuscript.

## Abbreviations

ECL: enhanced chemiluminescence
ER: endoplasmic reticulum
IBD: inflammatory bowel disease
LPS: lipopolysaccharides
PMSF: phenylmethane sulfonyl fluoride
TLR: Toll-like receptor
TUDCA: tauroursodeoxycholic acid
